# Child Home Care Allowance and the Transition to Second- and Third-Order Births in Finland

**DOI:** 10.1007/s11113-017-9437-1

**Published:** 2017-05-15

**Authors:** Anni Erlandsson

**Affiliations:** Swedish Institute for Social Research, Stockholm University, Universitetsvägen 10F, 10691 Stockholm, Sweden

**Keywords:** Cash-for-care, Family policy, Fertility, Finland, Home care allowance, Parity-specific

## Abstract

Using register data from the Finnish Census Panel, this paper studies the relationship between the use of the child home care allowance and second and third births among women aged 20–44 in Finland during the period 1992–2007. Discrete-time event-history analysis is applied to examine (i) whether women taking up the child home care allowance while their previous child was under the age of 3 have a higher risk to proceed to subsequent childbearing, (ii) whether these women proceed to a further birth more quickly, and (iii) whether the risk to proceed to a subsequent birth is related to educational level. The results show that women using the allowance have a higher risk of having a second and a third birth than women not using it. The risk of having a second birth is higher than that of having a third birth. Also, women using the allowance get their subsequent child sooner than women not using the allowance. No large educational differences in the effect of allowance use are found for second or third births.

## Introduction

Although Finland, as the other Nordic countries, has higher fertility than many other Western countries, its fertility rate is below the replacement level [defined as a total fertility rate (TFR) 2.1]. Therefore, Finland, like many of its Western counterparts, will face problems with an aging population—accompanied by a decrease in the share of people in working ages implying future difficulties in maintaining social services associated to a welfare state. Still, the relatively generous family policies, including paid parental leaves and publicly subsidized childcare, are likely to be, at least partly, a reason for Finland not being one of the European countries with lowest-low fertility (defined as a TFR at or below 1.3) (Adsera 2004; Brewster and Rindfuss 2000; Neyer 2005; Rønsen 2004).

Child home care allowance is an important component of Finland’s family policy. It is a benefit paid to a parent with a child under the age of 3 who does not use publicly financed childcare services. The purpose of the allowance is to increase parents’ flexibility in combining paid work and childcare as well as to allow them to spend more time with their children. The policy may, however, also lead to increased childbearing. Although the policy was intended to be gender neutral, the allowance is used almost exclusively by mothers. Hence, mothers’ labor market attachment is reduced which may decrease the opportunity costs of having another child.

Based on a 10% sample from population registers, this paper explores the relationship between second (third) birth and the uptake of the home care allowance in Finland during the period of 1992(3)–2007. This study examines whether the users of the allowance have a higher risk of proceeding to subsequent childbearing—second and third births—than those not using the allowance. The study also looks at timing differences, i.e., whether the allowance users proceed to a subsequent birth more quickly than non-users. As both fertility patterns, i.e., transition to second and third births, and the use of home care allowance are assumed to differ by education, also potential differences by education in the effects of the allowance on subsequent births are investigated. The overall effects and the timing differences are studied separately. However, as selection effects may exist, the analysis cannot demonstrate any causality between allowance use and subsequent childbearing.

There has been an ongoing debate in family politics on how to increase fertility while not decreasing female employment. A lot of research has looked at fertility and labor market outcomes. This study focuses on the relationship between a specific family policy that involves a cash transfer, encouraging women to stay at home instead of being active in the labor market, and the transition to second and third births. To date, there is only one Finnish study (Vikat 2004) on this topic and it uses different measurements and data than this study. Therefore, this paper contributes to previous research on the relationship between a cash-for-care policy—as the allowance is often referred to as in the literature—and subsequent fertility as well as its timing. The study is also important in an international context, not least due to the allowance being a much debated policy in other Nordic countries as well (Duvander and Ellingsæter 2016).^1^ Moreover, the study is interesting from a theoretical point of view because it aims to advance the understanding of the interrelation between parents’, i.e., mothers’, decisions regarding family and work, as both further childbearing and allowance take-up imply staying at home rather than engaging in market work.

## The Finnish Context

Finland followed the trend typical of many Western European countries implying a high TFR after World War II and a subsequent decrease following the baby boom. The TFR has been relatively stable varying approximately between 1.6 and 1.9 since the mid-1970s (Official Statistics of Finland, OSF 2016). In Finland, fertility started to slowly increase in the late 1980s, shortly after the introduction of the child home care allowance. The increase continued during the 1990s recession which contrasts to the development in Sweden, the neighboring country, where the fertility rate decreased during the recession and has in general been more pro-cyclical (Andersson 2000). In 2010, Finland had a TFR of 1.87, and it had been increasing for 8 years in a row; but by 2015, the TFR had decreased to 1.65 (OSF 2016).

Finland belongs to the group of Nordic countries classified as universalistic types of welfare states (see Esping-Andersen 1990 for the terminology) with rather generous family policies. As in the other Nordic countries, dual-earner couples are the norm in Finland. Finnish parents receive paid maternity and parental leaves (generally about 90 and 70% of previous earnings, respectively, though with a ceiling for high income) that end when the child is approximately 9–10^2^ months. The leave is job protected (i.e., parents have the right to return to work after the leave). Also, all children under school age are entitled to municipal day care after the parental leave period. Since 2005, if a woman gets another child within a 3-year period, she keeps the level of parental allowances at the same amount as for the previous child (Haataja and Juutilainen 2014), which may reduce the economic obstacles of staying at home longer and provide an incentive for having a subsequent child (sooner).

The Finnish child home care allowance was instituted already in 1985, being then unique to Finland. The allowance has since then been a politically debated topic mainly as it deviates from the dual-earner family model and also because the positive aspects of homecare are being contrasted with institutional childcare (e.g., Hiilamo and Kangas 2009). Consequently, there were changes in the policy in the 1990s as the amount of the benefit was reduced. Regardless, the home care allowance has turned out to be very popular, and almost 90% of families with a child born in the 2000s have used it (Haataja and Juutilainen 2014). Consequently, the share of (small) children in publicly financed day care is relatively modest in comparison to other Nordic countries (Sipilä et al. 2010).^3^

The home care allowance is taxable income, can only be received for a child below the age of 3 and cannot be used simultaneously with full parental or adoption benefits (for the same child). The allowance consists of two parts: the basic flat rate allowance (341 euros per month in 2016) is given separately for each eligible child, and a means-tested amount (at maximum 183 euros per month in 2016) which depends on the income and size of the family (Social Insurance Institution of Finland 2016). The allowance also includes a sibling supplement: 102 euros in 2016 for each child under the age of 3 and 66 euros for older children below school age who are cared for in a similar fashion. Some municipalities, but not all, provide supplements to the allowance, which is said to generate inequalities and unpredictability among families with young children, and some of the municipalities require those receiving the supplement to have a job to return to (Sipilä et al. 2010, p. 49). Yet, the allowance is a smaller amount than the parental leave allowance or a salary from full time work, and it is not enough to cover the living costs for a family. Therefore, the allowance may strengthen a traditional gendered division of labor which implies a caring mother and a breadwinner father in contrast to a dual-earner/dual-carer model.

In almost all (97%) families receiving child home care allowance, a parent stays at home to care for the child (Salmi et al. 2009), although someone else, e.g., a grandparent or a babysitter, could take care of the child instead. In Finland, the majority of small children have been minded at home, mainly by the mother, since 1990 (Sipilä et al. 2010, p. 50). The statistics from Finland show that over 90% of the home care allowance is used by mothers (The Social Insurance Institution of Finland 2014), and the rate is about the same in Norway where a similar policy has been in place since 1998 (Sipilä et al. 2010). Less than 10% of the fathers have used the allowance in Finland whereas over 50% of the mothers remain at home until the child turns 2 years (Sipilä et al. 2010, p. 81).

Although it is possible to take up the allowance even for short periods (1 month being the minimum), only about 29% of the users use it for less than 7 months and about 45% of the users for more than a year^4^ (The Social Insurance Institution of Finland 2014). Thus, a majority of the users rely on the allowance for quite a long time, and some of them very long. However, the share of parents with the longest periods of allowance take-up has decreased over time (Salmi et al. 2009). Mothers with temporary employment, lower income, or with several children receive the child home care allowance for the longest time (Salmi et al. 2009). Shared parental leave appears to be the only significant factor for a mother to not take up the allowance: mothers sharing parental leave with the father are found to be almost twice as likely as others not to use any child home care allowance when controlling for mother’s age, education, status in employment, and income (Salmi et al. 2009, p. 39).

## Theoretical Background and Previous Research

How to organize the care of children in relation to employment is an important decision that families encounter. The theory of the allocation of time by Becker (1965) provides a model for the decision-making process within the family in regard to the division of labor among household members. The theory implies that what is best for the household, i.e., the family, is best for the individual members. This implies that the household members who are relatively more productive in the labor market spend less time in consumption-related activities in comparison to other members (Becker 1965, p. 512). It applies also to the most common way of using the home care allowance: to allocate time to care for one’s child—which Becker regards as a time-intensive activity not productive in terms of earnings. Consequently, one may expect people with low income, i.e., typically those with low education and poor employment prospects, to be more likely to use the allowance.

Due to the lower opportunity costs of childbearing, low-income and low-educated women may be more likely not only to have children but also to proceed to subsequent childbearing than women with high education who often have higher income and human capital. According to Becker (1981), women’s higher educational attainment and labor market participation imply high opportunity costs of childbearing and this contributes to their decreased tendency to have children. Countries with high female labor market participation used to have low fertility but from the end of the 1980s the association is the reversed, especially at the macro level and also at the micro level (e.g., Adsera 2004). Yet, the micro-level evidence is not clear-cut (e.g., Andersson et al. 2014).

Institutional factors in the form of generous family policies, such as public childcare and home care allowance in particular, are found to be related to increased fertility (e.g., Adsera 2004). The reason is that they decrease the (opportunity) costs of having children and make it easier to combine family and career. Moreover, institutional factors, such as the availability or the lack of public childcare, may also affect preferences and decisions regarding childbearing and -rearing through the symbolic meaning of such family policies (Neyer 2005). The availability of public childcare may signal an opportunity to combine employment and parenthood thereby influencing childbearing decisions.

Ilmakunnas (1997), using Finnish survey data, finds quite a strong connection between the mother’s potential earnings—the value of mother’s time in the labor market—and the mode of childcare chosen at the turn of 1990s: the lower the potential wage, the more likely the mother is to stay at home with a young child. She also finds an increase in the level of home care allowance to increase the likelihood of choosing child home care rather than public childcare (Ilmakunnas 1997). In line with these findings, during 1991–1993—a time of increasing unemployment—the total number of day care places used by children aged 0–2 decreased by 26% in Finland, suggesting parents with poor employment prospects and those already unemployed to be more interested in cash in the form of the home care allowance than in day care (Sipilä and Korpinen 1998).

By using the allowance a mother can stay at home longer, and a long absence from the labor market may decrease one’s career prospects which could—by decreasing the opportunity costs of childbearing—make the effects of allowance take-up on childbearing more long-term. The economic loss of staying at home using the allowance instead of working is greater for mothers with higher earnings, and typically also higher education, than those with low wages. This applies not only to current but also to future income: with a steeper income curve loss of work experience leads to higher opportunity costs of having children. As the allowance is perceived as larger, in relative terms, for women with lower education and income, they are expected to be more likely to use it. Consequently, mothers with different educational levels who take up the allowance are assumed to differ in subsequent childbearing. If highly educated mothers decide to use the allowance, while being those least expected to use it, their allowance take-up may be driven by different reasons compared to low-educated mothers, possibly leading to divergent outcomes between the two groups of mothers.

Vikat (2004) examines the influence of women’s labor market attachment, earnings, and the use of child home care allowance on childbearing in Finland by using a 10% sample from a longitudinal register dataset representing the total female population in reproductive age 1988–2000. He shows that there is a positive educational gradient in the risk of first, second, and third birth. According to the findings, woman’s earnings and economic activity have a positive impact on the entry into motherhood and, to a lesser extent, on giving birth to a second child. Vikat (2004, p. 201) shows that there is a higher tendency to become a mother for women who are active in the labor market compared to non-active. While the study finds unemployed women to have the same risk of second birth as the employed, the unemployed have a higher third-birth risk. However, the parity-specific fertility trend in Finland is not greatly impacted by variations in the distribution of female population by activity and income (Vikat 2004, p. 201).

Vikat shows that the uptake of home care allowance is related to an increased third-birth risk but the risk of second birth does not differ by allowance use. The higher likelihood of those using the home care allowance to have a third birth may be mainly due to that the child- and family-oriented women take the allowance into consideration when planning to have a third child. Hence, the possibility to stay at home when children are young can be expected to be consistent with traditional family values (Vikat 2004, p. 203). Vikat (2004) argues that not finding any relationship between the allowance use and the risk of second birth may be due to that giving birth to a second child is the governing behavior for mothers with one child, and there may not be much space for the impact of the allowance use.

A lot of the existing research on child home care allowance and fertility comes from Norway where it is often referred to as the cash-for-care benefit.^5^ Aassve and Lappegård (2009), using register-based data, find that couples in which the mother has low-educational level and lower earnings have also the highest likelihood of using the allowance. Their findings indicate that the allowance use is positively related to birth timing, in particular proceeding to second birth within 2 years following the first birth. The results show considerable impact on third births, although to a smaller extent, whereas Lappegård (2010), also using Norwegian register data, finds that the implementation of the cash benefit has affected third births most.

Aassve and Lappegård (2010) find the use of cash benefits and subsequent birth timing to vary greatly by educational attainment. Highly educated women, especially those working within fields where establishing a career takes time and where the disadvantages of labor market absence are greater, will delay childbearing longer than those within other sectors or those with lower education (also see Lappegård and Rønsen 2005). Consequently, when these women finally have children they are likely to have them in a shorter time period because they have less reproductive time left. Also, Rønsen (2004) finds a significant positive effect of higher education on second births in Norway but not for Finland or for third births. Yet, there is research showing highly educated mothers in the Nordics to have higher second and third birth rates than mothers with lower education [e.g., Andersson et al. 2009 (Denmark, Finland, Norway, and Sweden); Vikat 2004 (Finland); Wood et al. 2014 (Norway)].

In contrast to Finland, in Norway before 2009, no entitlement for childcare existed before school age, meaning the age 6 (Holland 2011), and the demand for public day care seemed to exceed the supply (Lappegård 2010). Therefore, some of the allowance users in Norway may be more work-oriented and have higher education and income, than their Finnish counterparts if they use the allowance because they have not received a place in day care yet. This may imply lower rates of further childbearing among the allowance users in Norway in comparison to Finland.

Aassve and Lappegård (2010) argue that couples choose different strategies regarding work, childcare, and fertility. They find that the women using the home care allowance for the longest time possible progress more rapidly to a second birth than do others. This may be interpreted in such a way that the users of the allowance are a selected group oriented to home and family. Aassve and Lappegård (2010) conclude that although the introduction of the cash benefit policy seems to have contributed to a more rapid birth timing, it is unclear whether the policy has raised the overall fertility rates. If those who use the home care allowance are the same women for whom the opportunity costs of childbearing are low or who are more family-centered in their preferences, then one would expect no true effect of the allowance. Hence, any relationship between the allowance and fertility would be spurious, due to underlying common causes.

Also other studies, e.g., Sipilä et al. (2010), have pointed to the significance of parent’s preferences, their ideas of parenthood, and cultural values in affecting choices of what form of childcare to use, and whether or not to use the home care allowance. Preferences regarding childbearing are argued to be shaped by the socioeconomic status of the family of origin and number of siblings, and to be illustrated through one’s religiosity, union formation, and marital status (e.g., Rønsen 2004). Consequently, Ruokolainen and Notkola (2002), using Finnish survey data, find two-child mothers with traditional family values to be more likely to have third-child intentions. Yet, lifestyle preferences—including those regarding family and work—may be an outcome of fertility rather than a cause of fertility (Vitali et al. 2009, p. 436).

It is not only important to consider who uses the child home care allowance but also to think about the determinants of childbearing when considering the effects of the allowance on the fertility and its timing. Thus, the impact of institutional factors, culture, economy, and policy on childbearing decisions is important. Besides socioeconomic characteristics and individual preferences, these are also the main factors influencing the choice of childcare mode, i.e., the use of the allowance, which implies that the determinants of fertility and allowance take-up are closely related.

First, based on previous research, the hypothesis is that women using the allowance will be more likely to proceed to further childbearing, i.e., second- and third-order births, than those not using the allowance. Second, women who take up the allowance will proceed more quickly to further childbearing. The third hypothesis is that the effects of the allowance use on second and third births differ by educational level, i.e., the effect of the allowance is expected to be larger for low-educated women.

## Data

The dataset used in the analysis is the Finnish Census Panel retrieved from the Finnish population register with annual information covering the period 1991–2007. Yet, the analyses are performed during the period 1992(3)–2007 because the dependent variable, i.e., second (third) birth, relies on information from the previous year. Thus, inferring a first birth in year *t* means that there was no child in the household in year *t* *−* 1. Therefore, data are needed for the year prior to second birth and for 2 years prior to third birth. The dataset is compiled and coded by Statistics Finland and constitutes a 10% random sample of the population registered in Finland during this period.

Because the study examines the relationship between the use of home care allowance and fertility, and the allowance is mainly used by mothers, the unit of analysis is women. Thus, the population of the study is women aged 19–44 (within fertile ages) between 1991 and 2007, who had a first (and a second) birth during this period. The purpose is to compare the transition to second and third births between mothers who used the home care allowance at some point when the previous child was of eligible age to those who did not use it in order to answer the research question.

## Method

Discrete-time event-history analysis is applied in estimating the risks of second and third birth for users of the home care allowance in comparison to the non-users. Discrete-time is applied because time is measured in years (Allison 1984). The hazard rate *P*(*t*) in discrete-time is the conditional probability that an event—in this case the annual probability of birth of a second (third) child—will be experienced at a certain time (*t*) by a certain individual given that it has not been experienced earlier by that individual. The discrete-time logit regression model applied here, takes the following form:

$$\log \left( {\frac{P(t)}{1 - P(t)}} \right) = a(t) + \beta_{1} x_{1} + \beta_{2} x_{2} (t),$$

where *α*(*t*) is a set of constants varying by time, *β*_1_ is a vector of coefficients for time-constant control variables (e.g., age of mother at previous birth and its quadratic term), and *β*_2_ is a vector of coefficients for time-varying control variables (e.g., time since previous birth, use of home care allowance, marital status, and educational level). The annual probabilities are combined in a way that allows women to contribute to the risk each year, while removed from the risk pool after childbirth.

Because patterns of fertility differ by parity, the models for second and third births are calculated separately. Thus, there are two time processes: transition to second birth and transition to third birth. For second (third) birth, the time variable is time since first (second) birth, i.e., time starts in January the year after the first (second) birth for a woman aged 20(21)–44 years, and stops at the event, i.e., second (third) birth. Censoring occurs either at the age of 45, death, emigration, or at the end of the period studied, i.e., year 2007, whichever comes first.

Four main models were fitted to analyze the transitions to second and third births. Model 1 includes years since the previous birth and the use of home care allowance. It is applied mainly to see if there are differences in the risk of birth between the two groups—mothers not using the allowance and mothers using the allowance while the previous child was under the age of 3—but also to analyze the timing of subsequent birth. Model 2 adds a number of control variables, i.e., age and age^2^ of the mother at previous birth, marital status, educational level, and calendar year.

Model 3 also includes an interaction between years since the previous birth and allowance use, and it is applied in order to see if the groups differ in the timing of the next birth. Model 4 is similar to model 2 but additionally includes an interaction between the allowance use and educational level to study the differences in the effects of the allowance by education. Due to the large data size, a considerable part of the estimates becomes significant. For this reason, significance is shown only if the estimates are significant at or below the level of 1%.

## Variables

The time variable measures duration, i.e., time since previous birth, as explained in the method section. The main independent variable is a binary dummy indicating whether the mother received the home care allowance (when the previous child was eligible for it) while being at risk of second or third birth, respectively. For the risk of second (third) birth, the variable is zero as long as the mother did not receive the allowance for the first (second) child, and one from the year the allowance was received. If the allowance was not received by the time the previous child turned three (the upper age limit for usage), the variable took the value zero for the entire spell at risk. If the allowance was received, the variable was one starting from the year in which it was used until the end of the spell at risk. Thus, this variable measures whether the mother used the benefit (by time *t*) for the previous child during the eligible years. Being a time-varying variable on whether the allowance has ever been used during eligibility, this specification differs from one in which the variable would take the value one only in the years of actual usage. The latter would assume an immediate fertility effect during the eligible years and is thus less plausible.

The dependent variable—whether one gets a second or a third child or not—is measured by comparing the number of children in the household across years, thus assuming that the woman does not have children outside the household. This means inferring a birth occurring only if a new child appears in the household in year *t* + 1. The number of children in the household is measured the last day of each year. Hence, the exact date (day and month) of the births are not included in the data. In order to reduce potential bias, only a child under the age of 3 in the household is considered as a new child as there is no such information for younger ages in the data.^6^

Table 1 shows the number of mothers and person years in the analysis together with the mean and standard deviation for age at previous birth and calendar year. Table 2 shows the descriptive statistics for the other variables as a percentage of person years. 59,715 (37,359) mothers, i.e., about 87.5 (93.9) % of the mothers in the study who are under a risk of a second (third) birth have at some point used the home care allowance while their previous child was of eligible age (not shown in the tables).

**Table 1:** Mean and standard deviation for mother’s age at previous birth and calendar year, person years, and number of mothers in the analysis for second and third birth

	Second birth		Third birth	
	Mean	SD	Mean	SD
Age at previous birth	27.68	5.20	29.62	4.42
Calendar year	2001.02	4.23	2002.43	3.50
*N* mothers	68,217		39,795	
*N* person years	268,594		204,682

**Table 2:** Distribution of years of exposure at the different levels of variables applied in the models for second and third birth separately

	Second birth (%)	Third birth (%)
Use of home care allowance		
No	19.80	9.89
Yes	80.20	90.11
Civil status		
Non-married	51.57	32.80
Married	48.43	67.20
Educational levels		
No secondary education	15.41	11.40
Secondary education	44.27	42.28
Lower tertiary education	30.31	34.62
Upper tertiary education	10.01	11.70

Percent within each category

Sociodemographic characteristics such as mother’s age at previous birth, marital status, educational level, and calendar year are adjusted for in the analyses. These control variables represent factors that may influence both fertility and the use of home care allowance. The age of the mother at previous birth is a time-constant covariate measured in years and it is also measured non-linear, by additionally including a quadratic age term. The variable indicating marital status is annually time-varying and is measured as zero for non-married and one for married. Thus, the non-married category comprises single, those living in a non-marital union as well as divorced and widowed. Calendar year is a time-varying covariate measured in years.

Education is also a time-varying variable comprising four educational groups: no secondary education, secondary education, lower tertiary education (including post-secondary non-tertiary education and lower level tertiary education) and upper tertiary education (including higher level tertiary education and doctorate or equivalent level education). This categorization represents the structure of the Finnish educational system (Statistics Finland 2011).

## Results

**Fig. 1:**
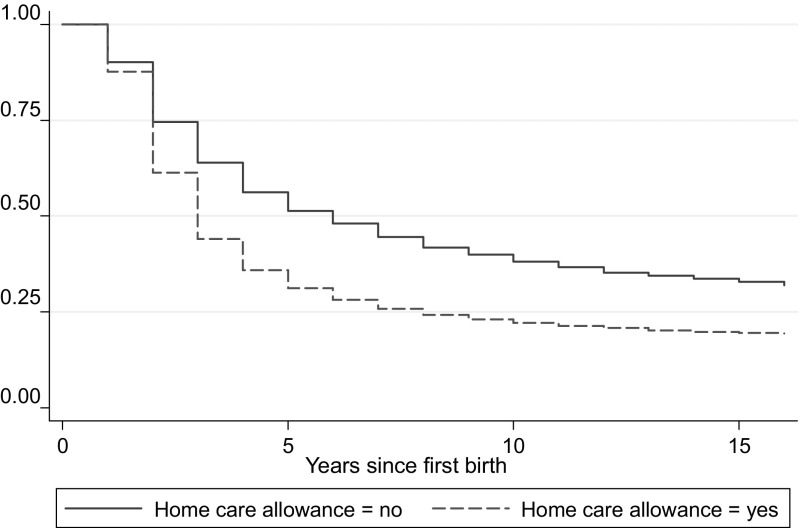
Kaplan–Meier survival estimates: second birth

**Fig. 2:**
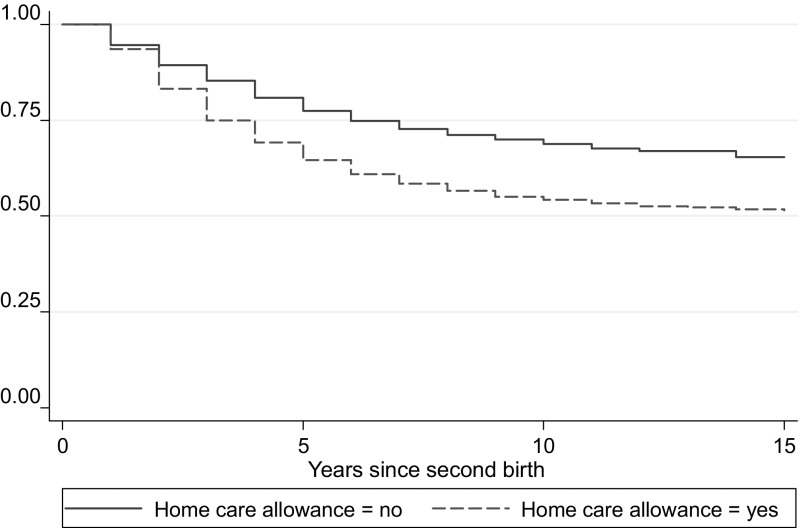
Kaplan–Meier survival estimates: third birth

Women using the child home care allowance have a higher risk of having a second and a third birth than women not using the allowance. The gap between the groups remains throughout the years, as shown in the Kaplan–Meier survival estimates in Figs. 1 and 2. To clarify, the event illustrated in Fig. 1 (2) is surviving from not having a second (third) birth during a certain time since first (second) birth.

Table 3 shows the results of the logit regression as odds ratios, relative risks, of birth separately for second and third births. The baseline odds are shown in the table in order to better explain the concept of odds in this analysis. Comparing the odds ratio with the baseline odds provides a clearer picture of the size of the effect (Buis 2012, p. 166). The baseline odds help to explain how high the risk of birth in the baseline is, meaning the reference group, which, for example, in Model 1 is women not using the allowance in the first year since the birth of the previous child. The baseline odds refer to the odds in the baseline category for each control variable, i.e., when all control variables in the model are zero, the value is the baseline value (Buis 2012, p. 166). Other estimates show the multiplicity of how much lower or higher the risk is when comparing with for instance those using the allowance or the second year to the reference group. In Model 1 for the second birth, the baseline odds for women not using the home care allowance (the reference group) and who had a second birth during the first year after having their first child were 0.084; among those who used the allowance, the odds were 1.74 times, or 74%, higher. Thus, the odds change from 0.084 to 0.146 (1.738 * 0.084 = 0.146), from 0.243 to 0.423 (0.084 * 2.895–0.084 * 2.895 * 1.738) in the second year, and from 0.218 to 0.380 (0.084 * 2.601–0.084 * 2.601 * 1.738) in the third year.^7^

**Table 3:** Relative risks (odds ratios) of birth for women of ages 20–44 in Finland 1992–2007 separately for second and third birth

	Second birth				Third birth			
	Model 1	Model 2	Model 3	Model 4	Model 1	Model 2	Model 3	Model 4
Years since previous birth								
1	1	1	1	1	1	1	1	1
2	2.895**	2.998**	1.989**	2.998**	1.764**	1.784**	1.049	1.783**
3	2.601**	2.823**	1.659**	2.823**	1.550**	1.591**	0.845	1.590**
4	1.555**	1.769**	1.392*	1.769**	1.200**	1.242**	0.956	1.242**
5	1.050	1.239**	0.974	1.238**	1.031	1.069	0.755	1.069
6	0.763**	0.911*	0.702**	0.910*	0.856**	0.883*	0.599*	0.883*
7	0.675**	0.808**	0.783*	0.808**	0.613**	0.625**	0.476**	0.625**
Use of home care allowance								
No	1	1	1	1	1	1	1	1
Yes	1.738**	1.706**	1.240**	1.881**	1.677**	1.505**	1.047	1.949**
Mother’s age at previous birth		1.026**	1.027**	1.026**		0.905**	0.905**	0.905**
Mother’s age at previous birth^2^		0.996**	0.996**	0.996**		1.000	1.000	1.000
Marital status								
Non-married		1	1	1		1	1	1
Married		2.263**	2.263**	2.264**		1.756**	1.755**	1.755**
Educational levels								
No secondary education		1	1	1		1	1	1
Secondary education		1.184**	1.184**	1.242**		0.983	0.983	1.292
Lower tertiary education		1.324**	1.324**	1.484**		0.972	0.972	1.208
Upper tertiary education		1.455**	1.456**	1.753**		1.137*	1.138*	1.638*
Years since previous birth * home care allowance								
1 year * home care allowance			1				1	
2 years * home care allowance			1.601**				1.759**	
3 years * home care allowance			1.834**				1.953**	
4 years * home care allowance			1.317**				1.322	
5 years * home care allowance			1.318**				1.450	
6 years * home care allowance			1.348**				1.516*	
7 years * home care allowance			0.998				1.336	
Calendar year^a^	No	Yes	Yes	Yes	No	Yes	Yes	Yes
Educational level * home care allowance								
No secondary education				1				1
Secondary education				0.949				0.753
Lower tertiary education				0.880				0.800
Upper tertiary education				0.806*				0.679
Baseline odds	0.084**	0.059**	0.077**	0.054**	0.042**	0.093**	0.131**	0.073**
*N* women	68217	68217	68217	68217	39795	39795	39795	39795
*N* observations	230133	230133	230133	230133	169419	169419	169419	169419
Log likelihood	−104198.36	−99994.924	−99904.899	−99987.761	−44987.659	−43758.63	−43740.841	−43755.904
*Chi* ^2^	73764.89	71685.79	71991.29	71636.20	72200.60	68535.97	68503.81	68526.61
Df	8	29	35	32	8	28	34	31

Author’s own calculations based on the Finnish Census Panel

* *p* < 0.01, ** *p* < 0.001

^a^See “Appendix”

As Model 2 shows, the age of the mother at first birth is found to increase the risk of having a second child. Instead of increasing linearly, the effect of mother’s age is curvilinear—being first positive (>1), then it levels out and becomes negative (age^2^ <1). There is a clear difference between married and unmarried mothers, as the former have approximately 2.3 times as high a risk of having a second birth compared to unmarried mothers. Also, there is a positive gradient of education on the risk of having a second birth: women with upper tertiary education have 46% higher risk of having a second child than women with no secondary education.

According to the goodness-of-fit test (available by request from the author), Models 3 and 4 give an improved fit (*p* < 0.01) compared to Model 2 except for Model 4 for third birth. This means that adding the interaction between years since the previous birth and allowance use as well as the interaction between the educational level and allowance take-up provides a more complete picture.

**Fig. 3:**
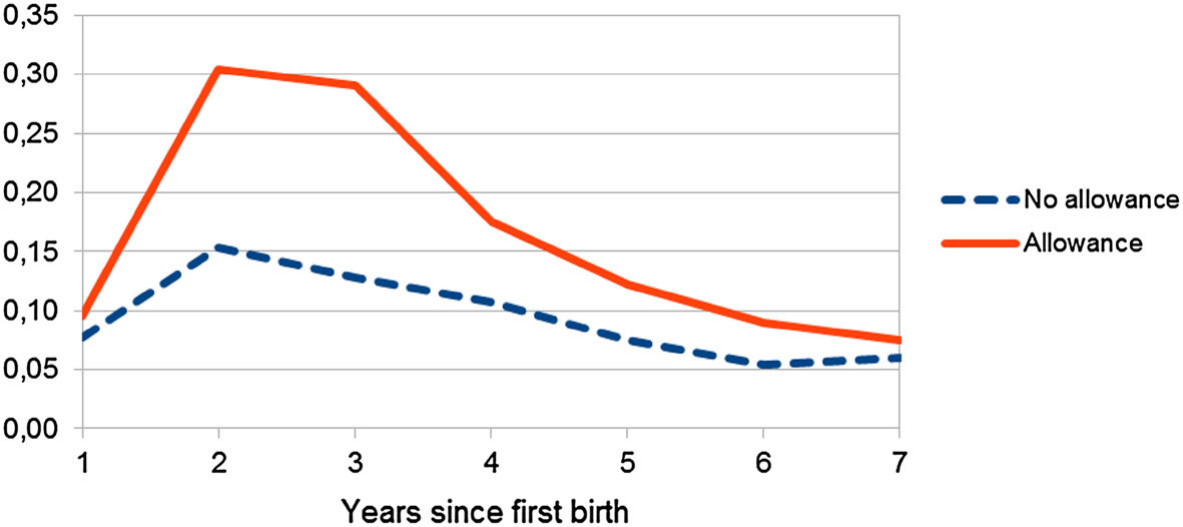
Predicted odds of second birth by allowance use. *Note* Based on Model 3 for second birth (Table 3) that includes the following covariates: years since previous birth, use of home care allowance, mother’s age at previous birth, mother’s age^2^ at previous birth, marital status, educational level, years since previous birth * home care allowance, and calendar year

In Model 3, the interaction between years since first birth and allowance take-up shows that women using the allowance have a higher risk of second birth each year (up to the sixth year) following the first birth than those not using the allowance. Figure 3 illustrates the interaction between years since first birth and allowance take-up based on Model 3. The predicted odds (0.153) for second births in the second year since the previous birth for mothers not using the allowance, for example, are calculated by multiplying the baseline odds (0.077) from Model 3 by the estimate for the second year since the previous birth (1.989). For mothers using the allowance, the predicted odds (0.304) for second births in the second year since the previous birth are calculated by multiplying the baseline odds by the estimates for the second year since the previous birth, for allowance use, and for the interaction of the two together (i.e., 0.077 * 1.989 * 1.240 * 1.601 = 0.304). The figure shows that the risk of second birth increases up to 2 years since the previous birth and starts then to decrease as time passes, thereby, illustrating that not using the allowance signals stopping behavior, also at longer durations.

Figure 5, which illustrates the interaction between allowance use and educational level in Model 4, shows that the risk of second birth seem to increase similarly with education both for mothers with allowance take-up and for mothers with no take-up. The predicted odds for allowance users presented in Fig. 5 are calculated by multiplying the estimates from Model 4 for baseline odds, allowance use, educational level, and the education interaction together (for non-users, the estimates for baseline odds are multiplied only by educational level). However, as shown in Model 4 in Table 3, the results for the interaction between allowance take-up and educational level for second birth are (mostly) not significant.

The results for third birth are very similar to those for second birth, though mostly smaller in extent. As Model 1 shows, women using the allowance have approximately 1.7 times as high a risk of having a third child as those not using the allowance. Model 2 shows that, unlike for the second birth, mother’s age at previous birth decreases linearly the risk of having a third birth, i.e., the older the mother when having her second child, the lower the risk for her to have a third child. For marital status, the pattern is similar for third birth but the estimate is not as strong as for the second birth. Married mothers are found to have 76% higher risk of having a third birth than unmarried mothers. In regard to educational level and the risk of a third birth, the pattern differs somewhat from the one for the second birth. Yet, also the risk of having a third birth is highest for the most educated women, i.e., those with upper tertiary education.

**Fig. 4:**
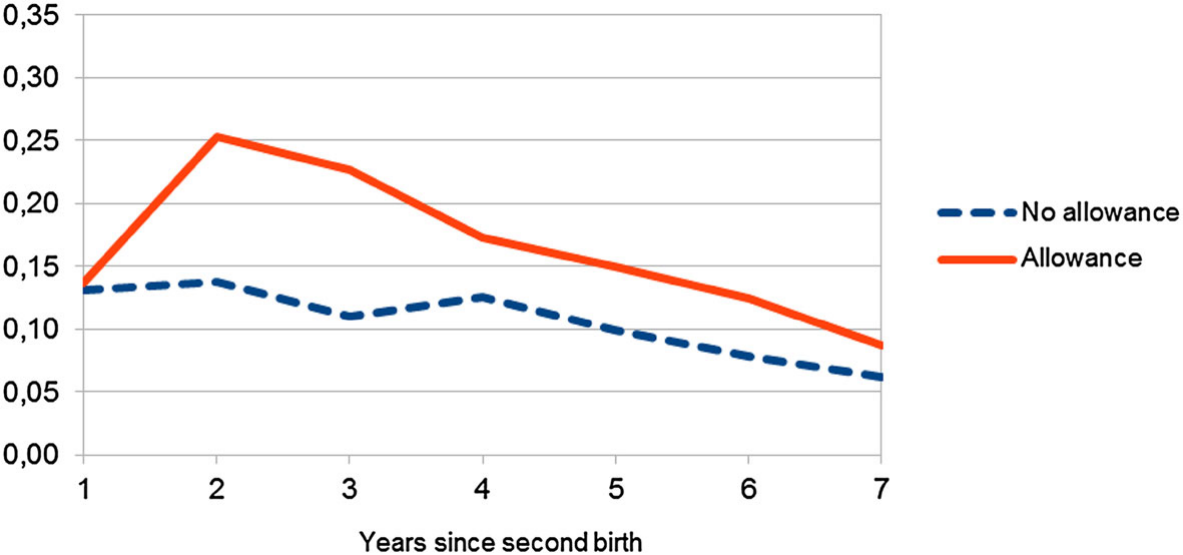
Predicted odds of third birth by allowance use. *Note* Based on Model 3 for third birth (Table 3) that includes the following covariates: years since previous birth, use of home care allowance, mother’s age at previous birth, mother’s age^2^ at previous birth, marital status, educational level, years since previous birth * home care allowance, and calendar year

**Fig. 5:**
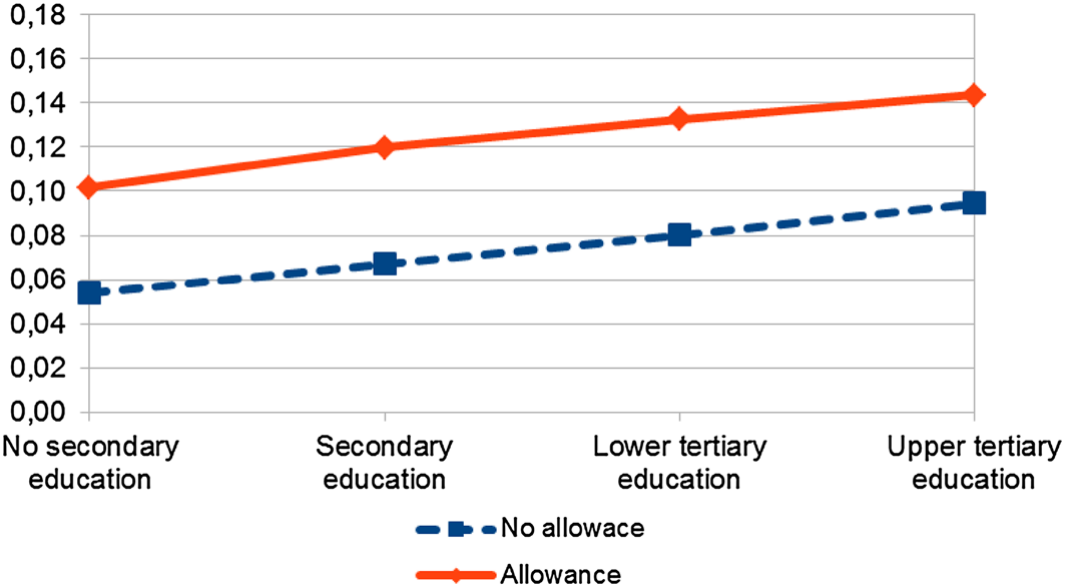
Predicted odds of second birth by allowance use and educational level. *Note* Based on Model 4 for second birth (Table 3) that includes the following covariates: years since previous birth, use of home care allowance, mother’s age at previous birth, mother’s age^2^ at previous birth, marital status, educational level, calendar year, and educational level * home care allowance

**Fig. 6:**
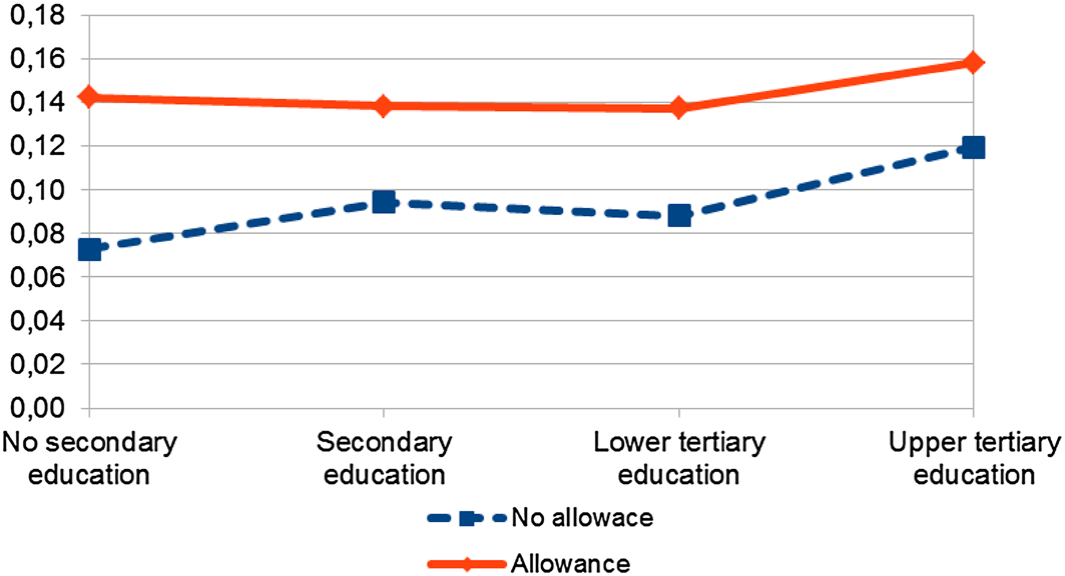
Predicted odds of third birth by allowance use and educational level. *Note* Based on Model 4 for third birth (Table 3) that includes the following covariates: years since previous birth, use of home care allowance, mother’s age at previous birth, mother’s age^2^ at previous birth, marital status, educational level, calendar year, and educational level * home care allowance

For third birth, the pattern for the interaction between allowance take-up and time since previous birth, as shown in Fig. 4, is quite similar for the one shown in Fig. 3 for second birth, i.e., the birth risk is highest in the second year for both the allowance users and non-users and then decreases over time. However, the results for the interaction between education and allowance use for third birth, as illustrated in Fig. 6, differ from those for the second birth in that the differences in the effect of the allowance between the users and non-users seem to decrease with education but the estimates are not significant. Therefore, as expected based on previous research, the low-educated seem most affected by allowance use. Contrary to what may have been expected, women with the highest education using the allowance seem to have the highest risk of second and especially of third birth as shown in Figs. 5 and 6. But due to the estimates (in Model 4 in Table 3) not being significant one should regard these results with caution and, therefore, the conclusion is that no large educational differences in the effect of allowance use on second or third births are found.

In summary, the main results for the second and third child are rather similar in that they show the uptake of the child home care allowance to have an effect on the risk of having an additional child. This is in line with the expectations. The results also reveal, as shown in Figs. 3 and 4, that timing varies by allowance use as there are differences in how soon women get their subsequent child that the control variables do not explain. There are no considerable differences in the risks of second and third birth by calendar year and, thereby, no clear period trends (the estimates for calendar year are presented in Table 4 in the “Appendix”).

## Discussion

Cash-for-care benefits have been a debated topic in Finnish politics since the second half of the twentieth century. The child home care allowance has been criticized for strengthening traditional gender roles by encouraging mothers to stay at home, thereby decreasing their labor market attachment. For this reason, and as the allowance is mainly used by women, it has often been labeled a “woman trap” (e.g., Hiilamo and Kangas 2009; Sipilä et al. 2010), contributing among others to an increase in their responsibilities at home, decreasing their retirements pensions, and widening gender wage gaps.

The results presented here imply that the use of the home care allowance has an effect on fertility by increasing it and speeding up the timing of second and third birth (though causality cannot be established). Thus, the results from this study support the hypothesis that women using the home care allowance have a higher risk of a second and a third birth, and they get a second and third birth sooner after the previous birth than those not using the allowance. The timing clearly matters: for the risk of second birth the difference between those using the allowance and those not using it is greatest in the third year since the first birth and then starts to decrease. This differs from previous findings from Finland which indicate the allowance take-up to be related only to a higher risk of third birth (Vikat 2004).

However, the previous Finnish study (Vikat 2004) studied a partly different time period, different variables (e.g., income), and different data than this study which may explain the differences in the results. When studying the effects of the home care allowance, Vikat (2004) looks at the risk of birth starting from January of the year when the youngest child turns 2 years, while in the current study the exposure to the subsequent birth risk starts in January of the year when the youngest child turns 1 year. Also, the variable measuring the allowance use is different compared to the one used here.^8^ These differences may to some extent explain why the findings (for the second birth) do not match those presented here.

The findings from Norway (Aassve and Lappegård 2009) indicate that the allowance use is positively related to birth timing, in particular to proceeding to a second birth within 2 years following the first birth. This positive relationship between the allowance take-up and second birth is in line with the results found here. Aassve and Lappegård (2010) find the child home care allowance to create a greater contrast in the timing of second birth for mothers with low education than for other groups. Although this paper does not study the differences in fertility timing by allowance use and education, the results for second birth show a slight negative gradient for education in interaction with allowance use, although the estimates for the interaction are mostly not significant. Yet, there appear no large differences in the effect of the allowance use on the risk of a second or a third birth by education. Considering the prevailing two-child family norm in Finland and the fact that most mothers use the child home care allowance, not finding large differences in the size of the effect by education between the users and non-users for second birth is not surprising.

Previous research illustrates the importance of considering institutional factors, social policies, culture, and economy when studying fertility trends. In the Nordics, the loss of income due to childbearing does not depict as high opportunity costs as in many other countries, partly due to large public sector, including the availability of public day care, job security, and generous parental leaves (Adsera 2004). Vikat (2004) suggests that the allowance may have decreased the economic impact related to unemployment and, therefore, reduced the influence of the recession on fertility behavior and childbearing plans in Finland. Also, no discernible effect of roller-coaster economic cycles on fertility was found in Finland (Vikat 2004) in contrast to Sweden—that is a very similar country in terms of the structure of the welfare state, except for the strong emphasis on income replacement in the Swedish parental leave system—where a pro-cyclical pattern of fertility with a positive relationship between women’s earnings and childbearing levels was found (Andersson 2000).

The differences observed in aggregated fertility between Finland and Sweden may to some extent be due to the higher third birth tendency that is positively connected to child home care allowance use in Finland while the risk of third birth declined about 40% in Sweden in the 1990s (Andersson 2000). Whereas Finland managed to maintain the essential features of the welfare state during the recession—regardless of reductions in some areas—the perception of the economic downturn as a passing phenomenon may have encouraged some women to take a break from employment exactly during the time when chances for career progress were unfavorable, perhaps also considering the option of prolonged leave for childcare connected to the home care allowance (Vikat 2004, p. 204). But as Vikat (2004) points out, while it could be the availability of the home care allowance that contributes to mothers fulfilling their plans of a subsequent child, it could also be the experience of staying at home focusing on childrearing itself that has an impact on the decision to have a subsequent child.

It is important to note the limitations of this study. Whether the child home care allowance is a cause for further, and faster, childbearing is an interesting question. However, the question of whether the policy itself influences fertility in Finland or whether the groups—those using the allowance and those not using it—differ in some way from each other cannot be answered by the current study. The results observed on childbearing differences by allowance uptake may be due to selection on preferences (Lappegård 2010) or, as it appears in Finland, those not using the allowance may be the select group. Although it is found that differences in the observed characteristics between the groups, i.e., mother’s age at previous birth and its quadratic term, marital status, educational level, and calendar year, do not explain the observed differences in fertility, a selection effect may exist.

The reason is that there may also be other observables—such as profession, income, career orientation, employment history, and information (e.g., income) about the partner—that affect childbearing decisions and outcomes. Due to data restrictions, these variables are not included in the analysis. Because the factors mentioned above are not available, this study cannot examine potential causality. Moreover, the effect of the introduction of the home care allowance policy on subsequent childbearing is not studied here because it requires data both before and after the policy was implemented.

Also, due to further data restrictions, the measure of allowance take-up used here is crude as it does not capture the length of the allowance use period. Therefore, the duration of allowance use and its effect on subsequent childbearing cannot be studied here. Yet, these are questions that would be interesting for future research, in addition to studying mothers using the allowance for both their first and second child. This may give more insight into how devoted the mothers are in terms of prioritizing family life and if the allowance is used for the spacing of a subsequent birth. Also, looking at the impact of allowance use on fertility by municipality, i.e., whether there are differences over time between municipalities that offer supplements to the allowance and municipalities that do not, would be interesting as it could bring one closer to answering the question of causality.

The evidence presented in this paper, i.e., that women using the allowance proceed more often and more quickly to subsequent childbearing than those not using it, is to a large extent in line with the previous findings and supports the claim that the allowance prolongs mothers’ stay out of the labor market. If a mother using the allowance gets another child right before her previous child turns 3 years and then uses the allowance for the newborn child until the child reaches the age of 3, she might stay out of the labor market for 6 years in a row. Such a long absence is very likely to have a negative impact on one’s working skills and social capital, as well as on the overall attractiveness in the labor market. To conclude, regardless of the importance of family time and parental care, in reality, as noted by Sipilä et al. (2010, p. 60) using the home care allowance might imply overburden on and isolation of mothers, creating a division of labor based on gender, exclusion of children from early childhood education, exclusion of mothers from the labor market, and life in poverty. However, a more gender equal uptake of the allowance could overturn many of its negative implications.

## Appendix

See Table 4.

**Table 4:** Estimates for calendar year for Models 2–4 in Table 3

	Second birth			Third birth		
	Model 2	Model 3	Model 4	Model 2	Model 3	Model 4
Calendar years						
1992	1	1	1	–	–	–
1993	0.897	0.900	0.897	1	1	1
1994	0.962	0.970	0.962	0.913	0.905	0.914
1995	0.980	0.990	0.980	0.952	0.946	0.953
1996	0.914	0.921	0.914	0.900	0.897	0.901
1997	0.868*	0.877	0.868*	0.871	0.871	0.872
1998	0.846*	0.857*	0.846*	0.860	0.861	0.861
1999	0.871*	0.882	0.870*	0.904	0.906	0.905
2000	0.872*	0.883	0.871*	0.930	0.930	0.930
2001	0.891	0.900	0.891	0.949	0.949	0.949
2002	0.871*	0.880	0.871*	0.951	0.951	0.951
2003	0.874*	0.884	0.874*	0.939	0.940	0.940
2004	0.872*	0.877	0.873*	0.960	0.959	0.961
2005	0.870*	0.875*	0.871*	0.985	0.985	0.986
2006	0.870*	0.876*	0.871*	0.997	0.997	0.998
2007	0.892	0.897	0.893	1.014	1.014	1.016
Baseline odds	0.059**	0.077**	0.054**	0.093**	0.131**	0.073**
*N* women	68217	68217	68217	39795	39795	39795
*N* observations	230133	230133	230133	169419	169419	169419
Log likelihood	−99994.924	−99904.899	−99987.761	−43758.63	−43740.841	−43755.904
*Chi* ^2^	71685.79	71991.29	71636.20	68535.97	68503.81	68526.61
Df	29	35	32	28	34	31

Author’s own calculations based on the Finnish Census Panel

* *p* < 0.01, ** *p* < 0.001

## Footnotes

1 For example, in 2016, Sweden abolished the home care allowance program that had been in place since 2008 (e.g., Duvander and Ellingsæter 2016).

2 Depending on how long before the expected birth date the mother started her maternity leave (it has to be started 30–50 days prior to the due date) and whether the father took the “daddy month” (that has been available since 2003). Prior to 2003, fathers could take a paternity leave of 18 days after the child was born but in 2003 the father was given 24 bonus days if he used the two last weeks of parental leave. In 2013, the “bonus month” for fathers was abolished and an extended paternity leave of 54 days (counting six work days per week), i.e., about 9 weeks, was introduced.

3 The share of 1–2-year-olds enrolled in day care was 40% in Finland in 2009, while the corresponding shares in other Nordic countries were 70–90% (NOSOSCO 2011). Only Norway had almost as a low share of children aged 1–2 enrolled in day care as Finland in 2000 (37 and 35%, respectively) but in 2009 this number had almost doubled in Norway (NOSOSCO 2011).

4 Until the child’s third birthday or the birth of another child.

5 See Rønsen (2004) for a more thorough comparison of the Finnish and Norwegian family policy systems, including the home care allowance.

6 It is very unlikely that a child under the age of 3 that is not the woman’s own child would live in her household as only a small proportion, less than 0.5% (European Community Household Panel 1996–2001), of children under the age of 18 do not live with their biological mother in Finland. Because it is such an extremely rare scenario this measure should not bias the results. Also, adopted children are eligible for child home care allowance, and they are regarded as any other children in the analysis because the data do not specify if a child is adopted. The number of adopted children below the age of 3 is rather low in Finland, e.g., 186–270 children below the age of 4 were adopted in a year during 1999–2003 (OSF 2010). Twins are excluded from the study due to data constraints.

7 Predicted probabilities (*P*), instead, can be calculated through the formula: *P* = odds/(1 + odds).

8 In Vikat’s (2004) study, the variable measuring allowance use that affects the childbearing risk in year *t* is determined by any take-up during year *t* *−* 1, whereas the variable constructed in this study is based on any take-up while the previous child was of eligible age, i.e., under the age of 3.

## References

[CR1] Aassve A, Lappegård T (2009). Childcare cash benefits and fertility timing in Norway. European Journal of Population.

[CR2] Aassve A, Lappegård T (2010). Cash-benefit policy and childbearing decisions in Norway. Marriage and Family Review.

[CR3] Adsera A (2004). Changing fertility rates in developed countries. The impact of labor market institutions. Journal of Population Economics.

[CR4] Allison PD (1984). Event-history analysis. Regression for longitudinal event data.

[CR5] Andersson G (2000). The impact of labour-force participation on childbearing behaviour: Pro-cyclical fertility in Sweden during the 1980s and the 1990s. European Journal of Population.

[CR6] Andersson G, Knudsen LB, Neyer G, Teschner K, Rønsen M, Lappegård T, Skrede K, Vikat A (2009). Cohort fertility patterns in the Nordic countries. Demographic Research.

[CR7] Andersson G, Kreyenfeld M, Mika T (2014). Welfare state context, female labour-market attachment and childbearing in Germany and Denmark. Journal of Population Research.

[CR8] Becker GS (1965). A theory of the allocation of time. The Economic Journal.

[CR9] Becker GS (1981). A treatise on the family.

[CR10] Brewster KL, Rindfuss RR (2000). Fertility and women’s employment in industrialized nations. Annual Review of Sociology.

[CR11] Buis ML (2012). Stata tip 107: The baseline is now reported. Stata Journal.

[CR12] Duvander AZ, Ellingsæter AL (2016). Cash for childcare schemes in the Nordic welfare states: Diverse paths, diverse outcomes. European Societies.

[CR13] Esping-Andersen G (1990). The three worlds of welfare capitalism.

[CR14] European Community Household Panel 1996–2001.

[CR15] Haataja, A., & V.-P. Juutilainen, (2014). Kuinka pitkään lasten kotihoitoa? Selvitys äitien lastenhoitojaksoista kotona 2000-luvulla [How long home care of children? A report on mothers’ child home care leave periods in the 2000s]. Työpapereita [Working papers] 58/2014. Helsinki: Social Insurance Institution of Finland

[CR16] Hiilamo H, Kangas O (2009). Trap for women or freedom to choose? The struggle over cash for child care schemes in Finland and Sweden. Journal of Social Policy.

[CR17] Holland, J. A. (2011). Cash-for-care, couple economic circumstances and marriage in Norway. Stockholm University Linnaeus Center on Social Policy and Family Dynamics in Europe (SPaDE) Working Paper 2011: 6, Stockholm University.

[CR18] Ilmakunnas S, Persson I, Jonung C (1997). Public policy and childcare choice. Economics of the family and family policies.

[CR19] Lappegård T (2010). Family policies and fertility in Norway. European Journal of Population.

[CR20] Lappegård T, Rønsen M (2005). The multifaceted impact of education on entry into motherhood. European Journal of Population.

[CR21] Neyer G (2005). Family policies in Western Europe. Fertility policies at the intersection of gender, employment and care policies. Austrian Journal of Political Science.

[CR22] NOSOSCO (2011). Social protection in the Nordic countries 2009/2010.

[CR23] Official Statistics of Finland, OSF. (2010). *Appendix table 1. Adoptions by age of child and by birthplace 1999–2010*. Helsinki: Statistics Finland. http://www.stat.fi/til/adopt/2010/adopt_2010_2011-06-01_tau_001_en.html. Cited 4 May 2016.

[CR24] Official Statistics of Finland, OSF. (2016). *Births*. Helsinki: Statistics Finland. http://www.stat.fi/til/synt/2015/synt_2015_2016-04-14_tie_001_en.html. Cited 23 January 2017.

[CR25] Rønsen M (2004). Fertility and public policies—Evidence from Norway and Finland. Demographic Research.

[CR26] Ruokolainen A, Notkola I-L (2002). Familial, situational, and attitudinal determinants of third-birth intentions and their uncertainty. Yearbook of Population Research in Finland.

[CR27] Salmi, M., Lammi-Taskula, J., & Närvi, J. (2009). *Perhevapaat ja työelämän tasa-arvo* [Family leaves and gender equality in working life]. Helsinki: Ministry of Employment and the Economy.

[CR28] Sipilä J, Korpinen J (1998). Cash versus child care services in Finland. Social Policy and Administration.

[CR29] Sipilä J, Repo K, Rissanen T (2010). Cash for childcare: The consequences for caring mothers.

[CR30] Social Insurance Institution of Finland. (2016). *Amount of the child home care allowance*. http://www.kela.fi/web/en/home-care-allowance-amount-and-payment. Cited 28 June 2016.

[CR31] Statistics Finland. (2011). *Educational level*. http://tilastokeskus.fi/meta/kas/koulutusaste_en.html. Cited 4 May 2016.

[CR32] The Social Insurance Institution of Finland. (2014). *Statistical yearbook of the Social Insurance Institution 2014*. Official Statistics of Finland. Social Protection.

[CR33] Vikat A (2004). Women’s labor force attachment and childbearing in Finland. Demographic Research.

[CR34] Vitali A, Billari FC, Prskawetz A, Testa MR (2009). Preference theory and low fertility: A comparative perspective. European Journal of Population.

[CR35] Wood J, Neels K, Kil T (2014). The educational gradient of childlessness and cohort parity progression in 14 low fertility countries. Demographic Research.

